# Dark-blood late gadolinium enhancement cardiovascular magnetic resonance for improved detection of subendocardial scar: a review of current techniques

**DOI:** 10.1186/s12968-021-00777-6

**Published:** 2021-07-22

**Authors:** Robert J. Holtackers, Caroline M. Van De Heyning, Amedeo Chiribiri, Joachim E. Wildberger, René M. Botnar, M. Eline Kooi

**Affiliations:** 1 Cardiovascular Research Institute Maastricht (CARIM), Maastricht University, PO Box 616, Maastricht, 6200 MD The Netherlands; 2 Department of Radiology & Nuclear Medicine, Maastricht University Medical Centre, Maastricht, The Netherlands; 3 School of Biomedical Engineering & Imaging Sciences, King’s College London, London, United Kingdom; 4 Department of Cardiology, Antwerp University Hospital, Edegem, Belgium; 5 Escuela de Ingeniería, Pontificia Universidad Católica de Chile, Santiago, Chile

**Keywords:** Cardiovascular diseases, Myocardial infarction, Magnetic resonance imaging, Late gadolinium enhancement, Myocardial scar

## Abstract

For almost 20 years, late gadolinium enhancement (LGE) cardiovascular magnetic resonance (CMR) has been the reference standard for the non-invasive assessment of myocardial viability. Since the blood pool often appears equally bright as the enhanced scar regions, detection of subendocardial scar patterns can be challenging. Various novel LGE methods have been proposed that null or suppress the blood signal by employing additional magnetization preparation mechanisms. This review aims to provide a comprehensive overview of these dark-blood LGE methods, discussing the magnetization preparation schemes and findings in phantom, preclinical, and clinical studies. Finally, conclusions on the current evidence and limitations are drawn and new avenues for future research are discussed. Dark-blood LGE methods are a promising new tool for non-invasive assessment of myocardial viability. For a mainstream adoption of dark-blood LGE, however, clinical availability and ease of use are crucial.

## Introduction

Late gadolinium enhancement (LGE), also sometimes referred to as late enhancement (LE) or delayed enhancement (DE), is a widely used cardiovascular magnetic resonance (CMR) technique to distinguish macroscopic scarring and myocardial infarction (MI) from normal myocardium. Since its initial validation against histology approximately two decades ago [1, 2], LGE has gained wide acceptance and is now considered the reference standard for the non-invasive assessment of myocardial viability. The clinical need for accurate scar detection was emphasized in the landmark study by Kwong et al. [3]. This study showed that using LGE, even small regions of scar tissue of only 2% of the mean left ventricular (LV) mass could be identified that were linked with a sevenfold increase in major cardiac events. Furthermore, the assessment of scar transmurality plays a major role in the prediction of the likelihood of regional functional recovery after revascularization [4], making LGE an important tool for image guided diagnosis, prognosis, and treatment planning.

The standard inversion-recovery (IR) LGE sequence with the inversion time (TI) set for myocardium nulling, however, has its limitations. Due to the often bright signal of the blood pool, blood may appear equally enhanced as adjacent subendocardial scar regions. As a result, these regions can be falsely interpreted as being part of the blood pool and therefore significantly reduce, or even completely obscure, the apparent scar volume. Scar tissue can also be mimicked by the blood pool signal in proximity of the subendocardium, resulting in false positive observations. Even though performing LGE at 20 min instead of 10 min post-injection intrinsically boosts scar-to-blood contrast due to contrast washout, this inefficient workaround is often not suitable for daily clinical practice.

Over the last 15 years, various novel “dark-blood” LGE approaches have been proposed to increase scar-to-blood contrast and improve subendocardial scar conspicuity. Most of these methods use additional magnetization preparation mechanisms to either suppress the blood pool signal partly (gray-blood techniques) or null the signal completely (black-blood techniques). These mechanisms include T_2_ preparation, magnetization transfer, and spin-locking in concert with the standard inversion pulse, and utilization of multiple inversion pulses. Similar effects, however, have also been achieved without using any additional magnetization preparation. As each approach utilizes a different contrast mechanism, a great variety in contrast between the normal myocardium, blood pool, and areas of myocardial enhancement can be achieved. This review aims to provide a comprehensive overview of current dark-blood LGE methods. For each method, the employed contrast mechanism and corresponding magnetization preparation scheme are illustrated, followed by a discussion on the findings in phantom, preclinical, and clinical studies. Finally, conclusions on the current evidence and limitations are drawn and new avenues for future research are discussed.

## Blood pool suppression techniques for LGE

### T_2_ preparation

**Fig. 1:**
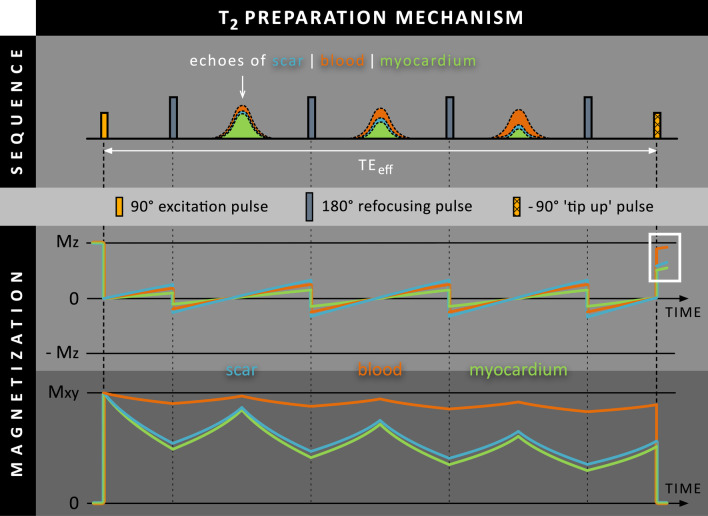
Schematic overview of the T_2_ preparation mechanism. A 90º radiofrequency (RF) pulse creates transverse magnetization that immediately starts decaying with a rate determined by the tissue-dependent T_2_ relaxation time. One or more 180º refocusing RF pulses are used to reduce signal dephasing caused by magnetic field inhomogeneities. After the effective echo time (TE_eff_), a -90º ‘tip-up’ RF pulse tips the remaining M_xy_ back towards the longitudinal axis, where the magnetization is stored again as M_z._ As the blood pool has a longer T_2_ relaxation time than scar tissue, the M_z_ blood will be higher than that of myocardium directly after T_2_ preparation (white rectangle)

T_2_ preparation can add additional contrast to the conventional heavily T_1_-weighted image based on the difference in T_2_ relaxation times of the normal myocardium and blood. Such a T_2_ preparation module starts with a 90º radiofrequency (RF) pulse that tips the longitudinal magnetization (M_z_) into the transverse plane (Fig. 1, top panel). For each tissue, the created transverse magnetization (M_xy_) will then start decaying with a rate given by the corresponding T_2_ relaxation time of that tissue, leading to significant signal loss in tissues with shorter T_2_ relaxation times, such as normal myocardium and areas of scarring (Fig. 1, bottom panel). One or more 180º refocusing RF pulses are applied to reduce dephasing caused by magnetic field inhomogeneities and to preserve M_xy_ of tissues with longer T_2_ relaxation times, such as the blood pool. After a specific time, called the effective echo time (TE_eff_), a -90º ‘tip up’ RF pulse tips the remaining M_xy_ back towards the longitudinal axis where the magnetization is stored again for subsequent imaging. The longer TE_eff_, the more T_2_ weighting was added to the available signal. As the blood pool has a significant longer T_2_ relaxation time than normal myocardium, blood magnetization will be higher than that of normal myocardium directly after T_2_ preparation (shown in white rectangle).

In 2005, Kellman et al. proposed their ‘multi contrast delayed enhancement’ (MCODE) approach that combined a two-beat phase-sensitive inversion-recovery (PSIR) LGE sequence with the acquisition of a T_2_-weighted image in the following third heartbeat [5]. Later in 2012, MCODE was validated in a cohort of 73 patients [6]. The extra T_2_-weighted image was able to separate the blood pool from both normal and infarcted myocardium, and thereby improving the detection of scar regions on the T_1_-weighted images (where both blood pool and scar areas appear bright).

Instead of acquiring an additional T_2_-weighted image, T_2_ preparation can also be incorporated into the LGE sequence itself to either suppress or completely null the blood pool signal. Two different forms can be distinguished based on the order of the T_2_ preparation module: those with T_2_ preparation before (T_2_ prep-IR) and those with T_2_ preparation after (IR-T_2_ prep) the inversion pulse.

#### T_2_ preparation before the inversion pulse

**Fig. 2:**
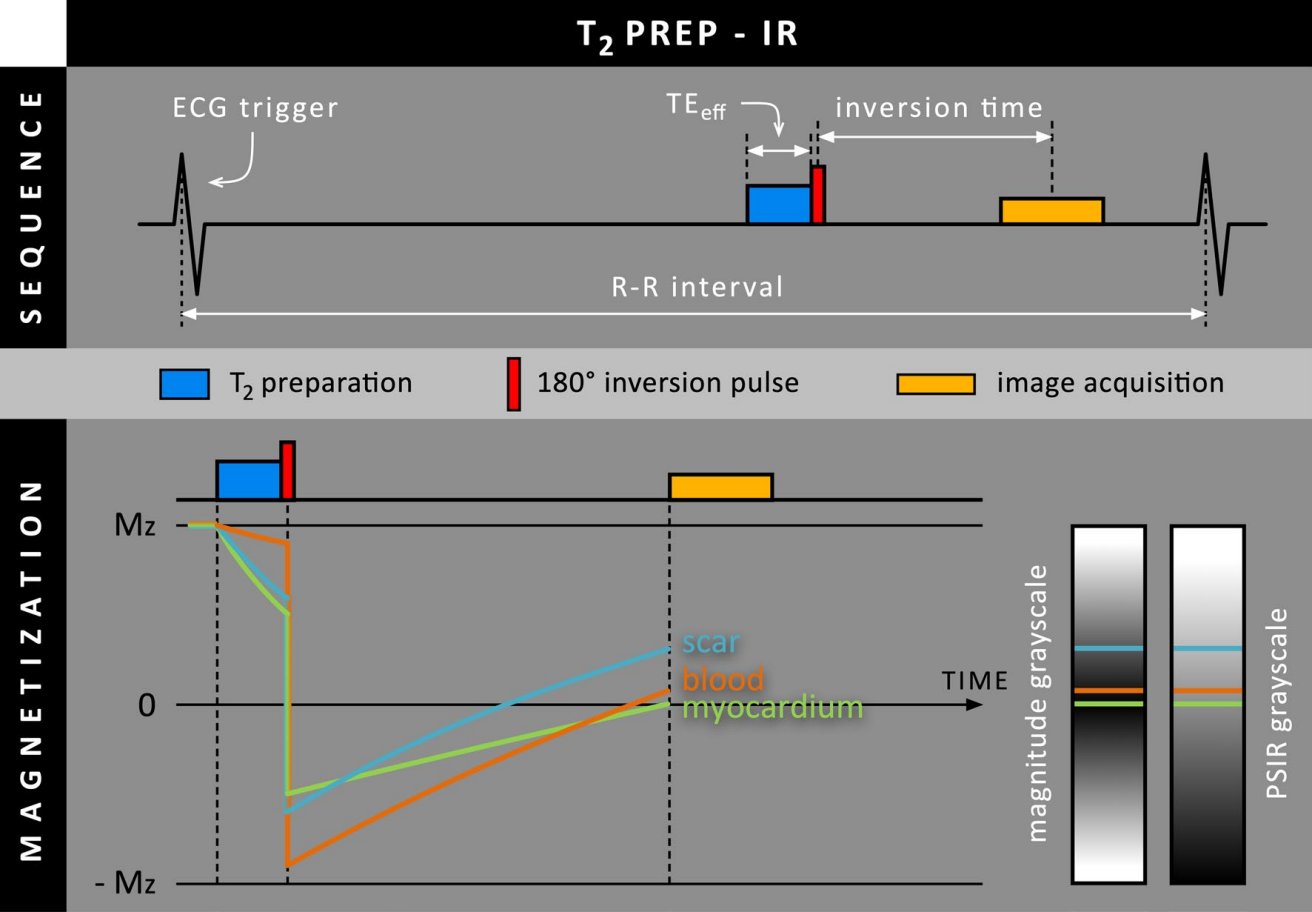
Schematic overview of the T_2_ prep-inversion recovery (T_2_ prep-IR) method, where T_2_ preparation is performed before the 180º inversion RF pulse. The effective echo time (TE_eff_) determines how long T_2_ preparation is performed before the remaining M_z_ is inverted. As blood is hardly affected by T_2_ preparation, the M_z_ of blood and scar are already separated before the 180º inversion RF pulse. Although they have similar T_1_ relaxation times, blood will have to start from a far more negative M_z_ level than scar tissue

In case of T_2_ prep-IR, the almost unaffected magnetization level of the blood pool after T_2_ preparation leads to a more negative magnetization level for blood after the inversion RF pulse compared to normal myocardium and scar tissue (Fig. 2). Although blood and scar have similar T_1_ relaxation times and recover almost equally fast, blood now starts from a more negative magnetization level than scar tissue, leading to increased scar-to-blood contrast compared to conventional LGE where both start from a similar magnetization level after the inversion RF pulse. Since blood has a much shorter T_1_ relaxation time than normal myocardium, blood recovers faster and can therefore catch up with the normal myocardium, allowing for simultaneous nulling of both tissues. By adjusting the TE_eff_ and TI, the blood pool appearance can range from being slightly suppressed to completely nulled.

In 2008, Liu et al. introduced their T_2_ prep-IR approach in a standard single-beat IR sequence [7]. Simulations for various combinations of TE_eff_ and TI were performed followed by an evaluation in five healthy subjects and nine patients with known MI. Contrast-to-noise ratio (CNR) measurements showed a 32% increase in scar-to-blood contrast compared to conventional LGE. While T_2_ prep-IR may be a valuable tool for improved detection and assessment of subendocardial scar tissue, it was mentioned that the per-patient optimization of TE_eff_ and TI is a potential pitfall.

#### *T*_*2*_* preparation after the inversion pulse*

**Fig. 3:**
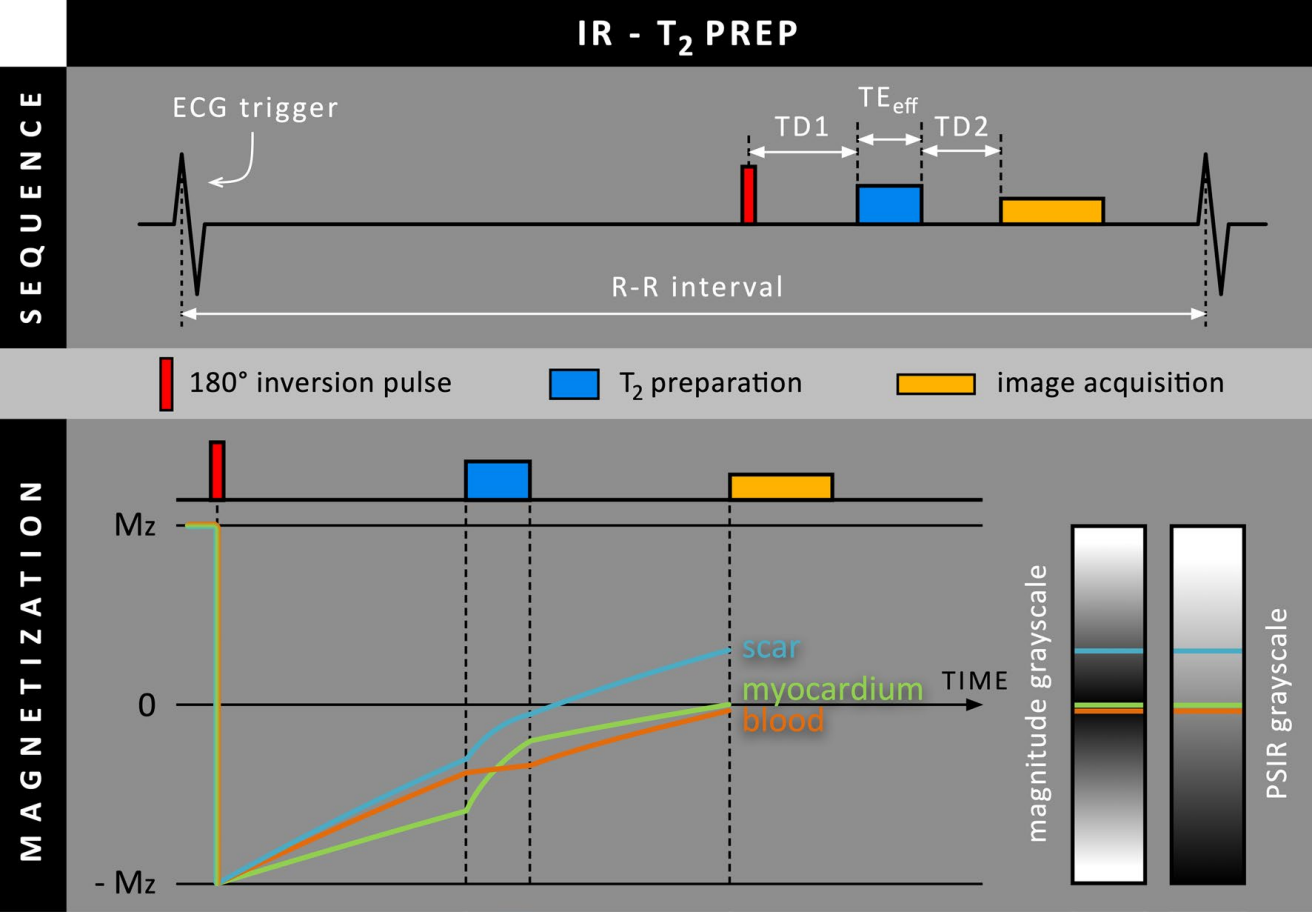
Schematic overview of the IR-T_2_ prep method, where T_2_ preparation is performed after the 180º inversion RF pulse. Note that the inversion time is split into two delay parameters (TD1 & TD2), separated by the T_2_ preparation module with duration TE_eff_. Due to the relatively long T_2_ relaxation times of scar and myocardium, their M_xy_ decreases quickly and thereby effectively ‘accelerates’ their M_z_ recovery during T_2_ preparation. As blood is hardly affected by T_2_ preparation, the M_z_ recovery of blood is effectively ‘paused’ during T_2_ preparation

In contrast to T_2_ prep-IR, T_2_ preparation can also be performed after the inversion pulse (IR-T_2_ prep**, **Fig. 3). The magnetization levels are then inverted first, after which they recover by T_1_ relaxation until T_2_ preparation starts. T_2_ preparation will then indirectly affect the rate at which the magnetization is recovering. Since normal myocardium and scar tissue have a relatively short T_2_ relaxation time compared to blood, their increased signal loss during T_2_ preparation will lead to a lower (negative) magnetization level after T_2_ preparation than it would have without T_2_ preparation. The recovery of normal myocardium and scar tissue have therefore effectively been accelerated during T_2_ relaxation. In contrast, the relatively long T_2_ relaxation time of blood results in far less signal loss during T_2_ preparation, leading to a largely maintained negative magnetization level after T_2_ preparation. As a result, the magnetization levels of blood and normal myocardium will cross each other during T_2_ preparation. Following T_2_ relaxation, both tissues will further recover based on their individual T_1_ relaxation times again. As blood recovers faster, the magnetization levels of blood and normal myocardium will cross again later. Instead of a single TI, two delay times have to be defined now: one between the inversion pulse and the T_2_ preparation module (TD1), and one between the T_2_ preparation module and image acquisition (TD2). By adjusting the two delay times and the TE_eff_, simultaneous nulling of the blood and normal myocardium can be achieved.

In 2016, Kellman et al. presented their IR-T_2_ prep method in a free-breathing PSIR sequence and combined it with respiratory motion-corrected averaging [8]. Simulations were performed to obtain LGE images with various degrees of blood pool suppression. Subsequently, their approach was evaluated in a cohort of 61 patients which showed that subendocardial MI was observed best when nulling the blood pool completely. Their CNR measurements, performed on a subset of 30 patients, showed increased scar-to-blood contrast compared to conventional LGE, however, at the cost of scar-to-myocardium contrast. Before image acquisition, a T_1_ map was required to obtain actual T_1_ relaxation times of both normal myocardium and the blood pool. After providing these times to the system, a custom ‘delta’ parameter is chosen which determines whether normal myocardium signal should be above or below that of the blood pool. The system then calculates TD1, TD2, and TE_eff_ using a strategy that sought to achieve the desired blood suppression while keeping TE_eff_ as short as possible to minimize signal loss.

In 2017, the same group compared this IR-T_2_ prep PSIR LGE approach to conventional bright-blood LGE in a larger cohort of 172 patients [9]. The IR-T_2_ prep approach found significantly more segments that exhibited LGE and allowed for increased confidence with regard to scar detection when nulling the blood pool. Additionally, 18 patients with no enhancement on bright-blood LGE images were found to have LGE on dark-blood images, 15 of whom had no known history of MI. However, no histological confirmation was available.

In 2018, Basha et al. proposed their IR-T_2_ prep approach for a free-breathing 3D acquisition without a PSIR reconstruction to reduce scan time [10]. Simulations and phantom experiments were performed showing the ability to simultaneously null the blood pool and normal myocardium. Based on the numerical simulations and phantom study, a contrast scout scan was developed that was used for all in-vivo imaging. For this scan, TE_eff_ and TD2 were kept constant (at 35 and 150 ms, respectively), while TD1 was sampled between 15 and 115 ms with 5 ms increments, yielding 21 images with different tissue contrast. This scan was performed prior in-vivo LGE imaging, analogous to a Look-Locker scan. Their IR-T_2_ prep approach was evaluated in nine infarcted swine and 42 patients. For both IR-T_2_ prep and conventional LGE, 22 out of 42 patients were found showing myocardial enhancement. Quantitative analysis in a subset of 17 patients and all swine showed increased scar/blood signal ratios for IR-T_2_ prep LGE compared to conventional LGE.

Also in 2018, the same group exploited the flexibility of their IR-T_2_ prep LGE approach by acquiring a suppressed (gray-blood) rather than a completely nulled blood pool (black-blood) using another set of parameter values [11]. Simulations were performed and a comparison between conventional LGE, black-blood IR-T_2_ prep LGE, and gray-blood IR-T_2_ prep LGE was carried out in 45 patients and five swine. Similar to the IR-T_2_ prep approach of Kellman et al., a T_1_ map was acquired prior LGE imaging to calculate the optimal imaging parameters. CNR measurements showed that although the black-blood approach outperformed gray-blood and conventional bright-blood LGE in terms of scar-to-blood contrast, scar-to-myocardium, and myocardium-to-blood contrast both decreased. In contrast, only gray-blood IR-T_2_ prep LGE achieved both increased scar-to-blood contrast and scar-to-myocardium contrast compared to conventional LGE. Furthermore, gray-blood LGE detected more scars compared to black-blood and conventional LGE. Subjective scores of the ability for localizing left-ventricular scar tissue and detecting papillary muscle scar was significantly improved for dark-blood LGE compared to both black-blood LGE and conventional LGE.

Although T_2_ preparation can be performed before or after the inversion pulse, both options have their pros and cons. By performing T_2_ preparation after the inversion pulse, an additional delay is created between the inversion pulse and T_2_ preparation module (Fig. 3). As a result, an additional third parameter, aside the T_2_ preparation duration (TE_eff_) and standard TI delay in T_2_ prep-IR, is available for IR-T_2_ prep to optimize contrast. On the other hand, however, the optimization process for the two delay parameters and TE_eff_ is more demanding. Additionally, performing the preparation after the inversion pulse limits the shortest inversion that can be set. Hence, such techniques may not work soon after contrast administration.

### Magnetization transfer preparation

**Fig. 4:**
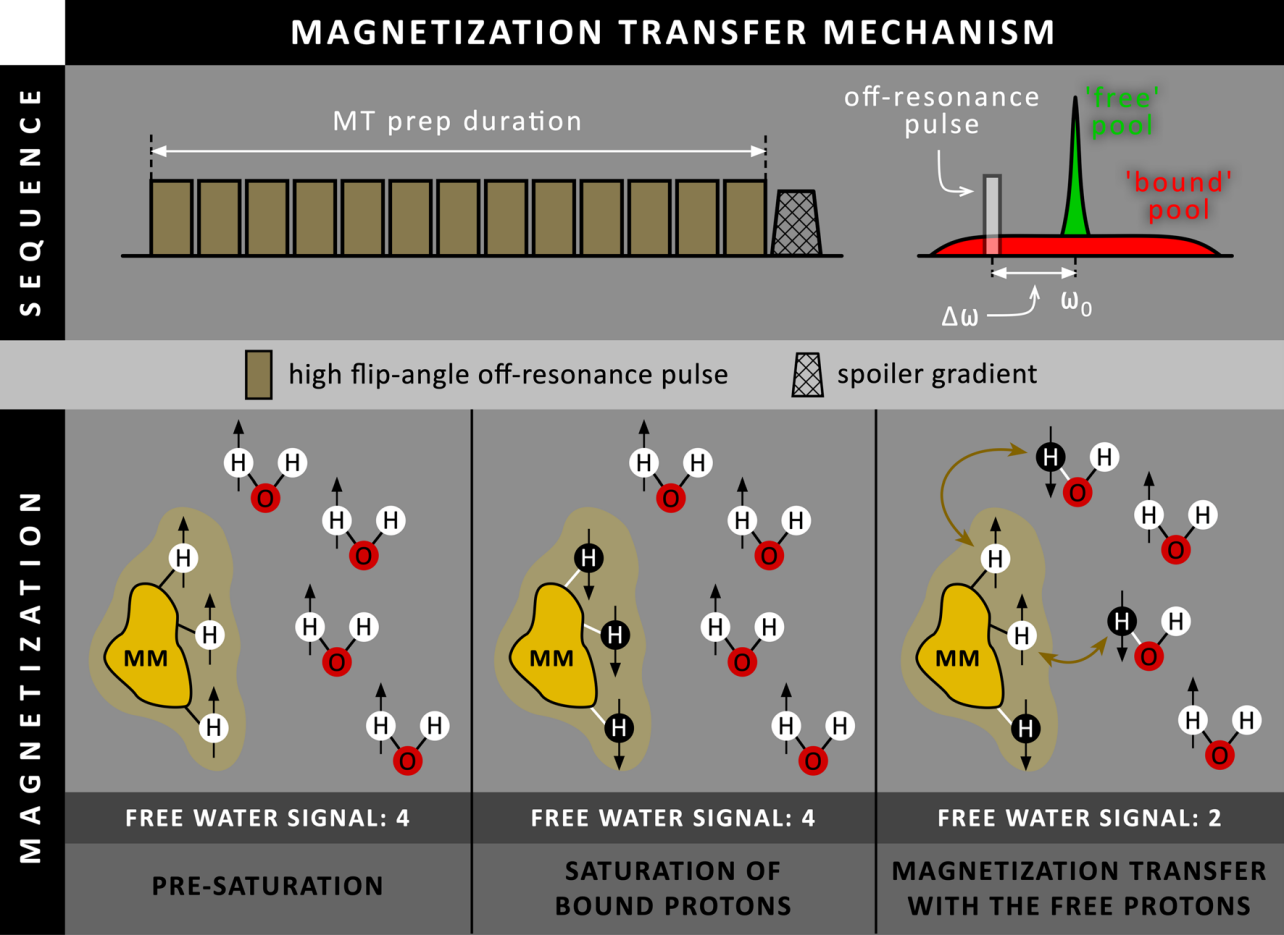
Schematic overview of the magnetization transfer (MT) mechanism. A series of high flip-angle off-resonance (Δω) RF pulses, followed by a spoiler gradient, are performed to selectively saturate the bound proton pool (macromolecules or MM). Since the bound proton pool and free proton pool (water molecules) are in continuous exchange with each other (curved arrows), magnetization is transferred from the free to the bound proton pool, leading to a decrease in net magnetization for the free proton pool and thus also in the detected signal. ω_0_ = Larmor frequency

**Fig. 5:**
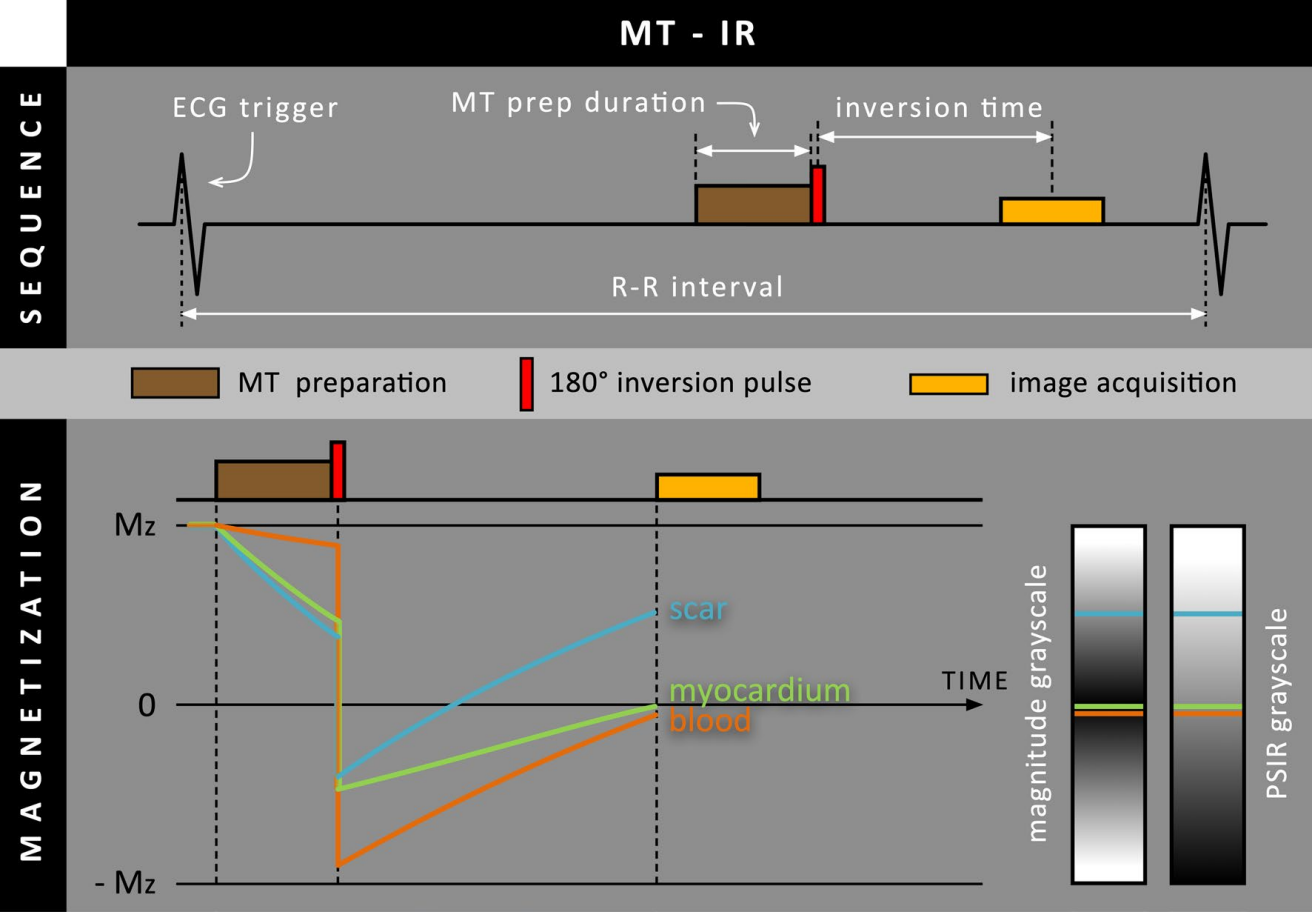
Schematic overview of the magnetization transfer (MT)-IR method, where MT preparation is performed before the 180º inversion RF pulse. The MT prep duration determines how long magnetization transfer is performed before the remaining M_z_ is inverted. As the blood pool is hardly affected by MT preparation, blood and scar magnetization levels are already separated before the 180º inversion RF pulse. Although they have similar T_1_ relaxation times, the blood pool will have to start from a far more negative M_z_ level than scar tissue

Another contrast mechanism that can be used for increasing scar-to-blood contrast in LGE is magnetization transfer (MT): a process in which magnetization is exchanged between protons existing in two different pools, the detectable ‘free’ pool and the ‘bound’ pool [12]. During MT preparation, the ‘invisible’ bound pool is selectively saturated using a train of high flip-angle (500–800º) off-resonance (600–800 Hz offset) RF pulses followed by a spoiler gradient, leading to a loss of net magnetization for the bound pool (Fig. 4). However, as the bound and free pool are in continuous exchange with each other, magnetization will be transferred from the free to the bound pool, leading to a magnetization decrease in the free pool and thus in the detectable CMR signal. When looking at the heart, blood primarily consists of a large free pool and negligible bound pool, resulting in a minimal signal drop during MT preparation. In contrast, normal myocardium and scar tissue consist of a sizeable bound pool, causing a significant signal drop during MT preparation. When performing MT preparation before the inversion pulse (MT-IR), the almost unaffected magnetization level of blood after MT preparation is inverted to a more negative magnetization level compared to the normal myocardium and scar areas (Fig. 5). Although blood and scar tissue have similar T_1_ relaxation times and recover almost equally fast, blood now starts from a more negative magnetization level than scar tissue, resulting in increased scar-to-blood contrast.

In 2015, Kim et al. proposed the ‘flow-independent dark-blood delayed enhancement’ (FIDDLE) method [13], designed as a modular approach to accommodate different magnetization preparation modules before the inversion pulse in a PSIR LGE sequence. In 2018, the performance of a FIDDLE variant, where MT was performed before the inversion pulse (MT-IR), was evaluated. [14]. First, a pilot study was performed in eight canines with induced MI to investigate the effects of MT preparation on the magnetization levels of both normal and infarcted myocardium, and blood pool. Using FIDDLE, the blood magnetization level was found below that of both normal and infarcted myocardium over a wide range of inversion times, therefore appearing black in the PSIR image (Fig. 5). The diagnostic performance of FIDDLE was assessed in 22 canines with histopathology as the reference standard, showing that the shape and contour of hyperenhanced regions on FIDDLE closely resembled those observed by histopathology (-0.1% bias). FIDDLE also showed increased sensitivity (96 vs 85%) and accuracy (95 vs 87%) for the detection of MI compared to conventional LGE. The clinical performance of FIDDLE was further evaluated in 31 patients, of which 20 had MI. FIDDLE was able to resolve cases with ambiguous conventional LGE images, clearly distinguishing between patients with and without hyperenhanced areas, with no differences between 1.5 T and 3 T. Although scar-to-blood contrast increased using FIDDLE, CNR measurements in 11 additional patients showed a 14% and 10% loss in scar-to-myocardium contrast on 1.5 T and 3 T, respectively, compared to conventional LGE. As the characteristics for MT preparation were optimized in the preceding pilot study, only the TI needed to be chosen using a Look-Locker sequence, which was set as long as possible while still fulfilling the prerequisite that the blood pool signal was below that of normal myocardium.

A T_2_ preparation variant of FIDDLE (T_2_ prep-IR), similar to the T_2_ prep-IR approach introduced by Liu et al. in 2008, was also exploited. However, T_2_ preparation was found to be inferior compared to MT preparation because of image artefacts (due to B_0_ and B_1_ sensitivity) and different levels of blood pool suppression in the right ventricle (RV) and LV (due to different T_2_ relaxation times) [15].

### Spin-lock preparation

**Fig. 6:**
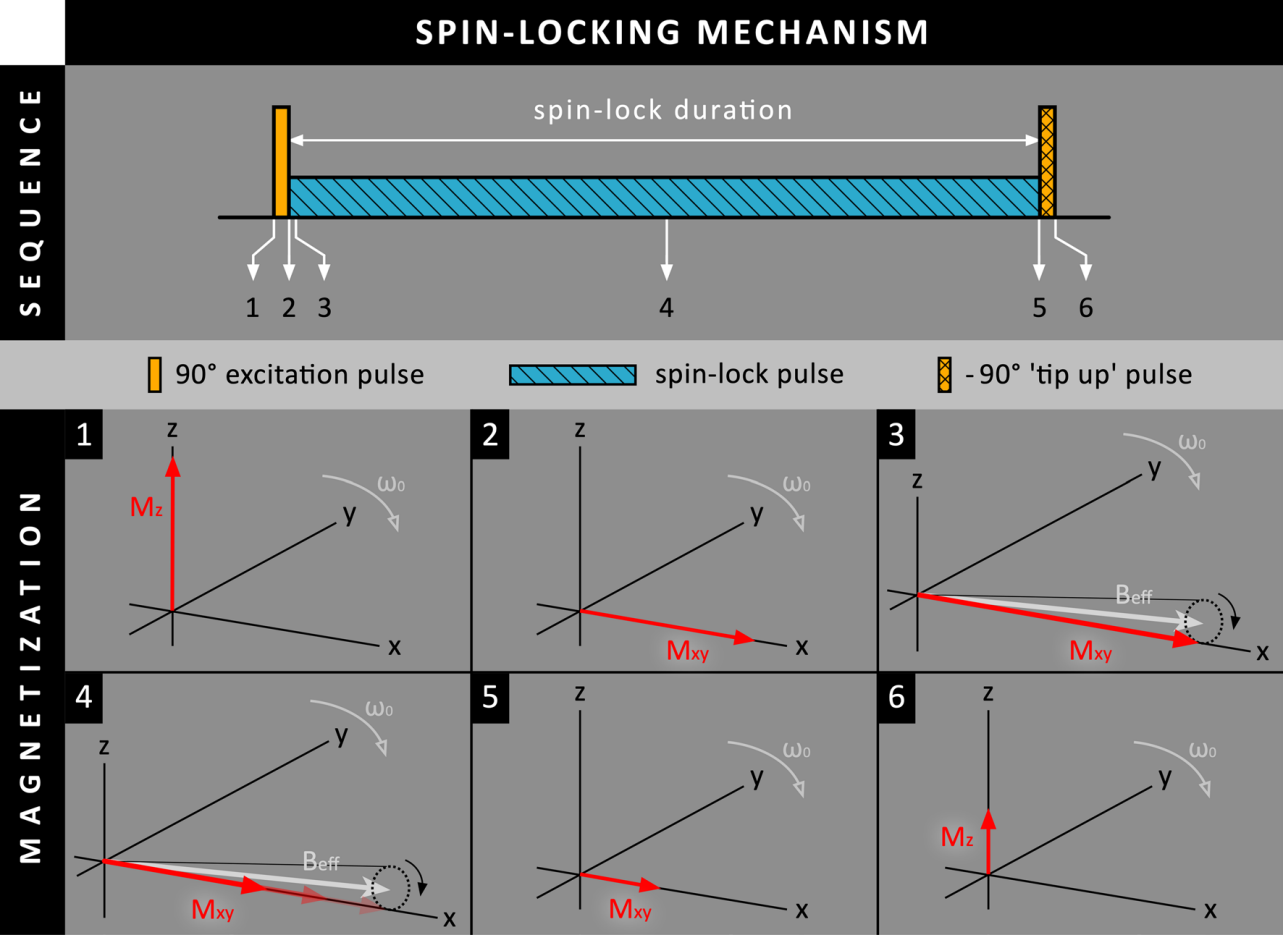
Schematic overview of the spin-locking mechanism. (1 + 2) A 90º RF pulse tips M_z_ into the transverse plane, creating M_xy_. (3) Directly afterwards, a continuous RF pulse is applied (nearly) parallel to M_xy_, creating the effective spin-lock field (B_eff_) (4). M_xy_ will now start rotating around B_eff_ in a narrow cone and is therefore ‘locked’. Instead of normal T_1_ and T_2_ relaxation, M_xy_ will undergo T_1_-rho relaxation. (5 + 6) A -90º ‘tip-up’ RF pulse tips the remaining M_xy_ back towards the longitudinal axis, where the magnetization is stored again as M_z_. The light gray arrows indicate a rotating frame of reference rotating with the Larmor frequency (ω_0_). Note that images 3 + 4 have been slightly enlarged for improved visualization

Besides T_2_ and MT preparation, a third contrast mechanism known as ‘T_1_-rho relaxation’ (T_1_ relaxation in the rotating frame) can be used to improve scar-to-blood contrast. In T_1_-rho CMR, a series of RF pulses are used to create a situation called ‘spin-lock’ (Fig. 6) [16]. Although T_1_ and T_2_ relaxation are still taking place, the magnetization is continuously disturbed by the RF pulse and cannot return to its equilibrium. Instead, the transverse magnetization now decays due to T_1_-rho relaxation, which relaxation times are longer than that of regular T_2_ relaxation times. In contrast to conventional T_1_ relaxation, the interactions between water and other molecules (such as the exchange and rotational correlation times) now have to be near the spin-lock frequency, instead of the Larmor frequency, for relaxation to occur. Since macromolecules, such as collagen, have rotational correlation times at the order of the spin-lock frequency of ~ 500 Hz, the T_1_-rho relaxation mechanism is highly sensitive to their interaction with water. Animal studies showed that infarct regions, which contained higher collagen fractions on histology, had significantly higher T_1_-rho relaxation times compared to normal myocardium [17]. Although T_1_-rho relaxation CMR on its own may be of interest as an endogenous contrast method, the use of spin-locking after contrast administration as additional preparation module for LGE has also been evaluated recently.

**Fig. 7:**
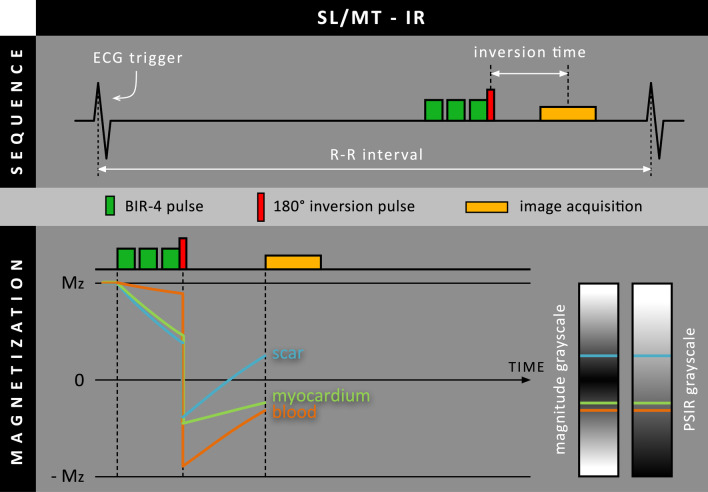
Schematic overview of the SL/MT-IR method, where both spin-lock (SL) and magnetization transfer (MT) preparation are performed using three consecutive adiabatic B_1_-insensitive rotation-4 (BIR-4) RF pulses before the 180º inversion RF pulse. As these BIR-4 RF pulses hardly affect the blood pool, in contrast to the scar tissue, blood and scar magnetization levels are already separated before the 180º inversion RF pulse. Although they have similar T_1_ relaxation times, the blood pool will have to start from a far more negative M_z_ level than scar tissue

In 2017, Muscogiuri et al. presented an LGE approach called ‘T(rho) and magnetization transfer and inversion recovery-prepared imaging’ (TRAMINER), where both spin-locking (SL) and MT are applied before the inversion pulse (SL/MT-IR) [18]. While a train of off-resonance MT pulses (typically 15–20) creates a ‘clean’ MT contrast in the MT-IR approach, TRAMINER uses three consecutive adiabatic B_1_-insensitive rotation-4 (BIR-4) pulses leading to a mixture of T_1_-rho and MT contrast (Fig. 7). These BIR-4 pulses led to attenuation of tissue magnetization while only minimally affecting the blood pool. Interestingly, in contrast to most other PSIR LGE techniques, TRAMINER uses an additional heartbeat for magnetization recovery in between the heartbeat with the image readout and the heartbeat with the PSIR reference readout (three heartbeats in total).

TRAMINER was evaluated in 40 patients with known or suspected prior MI [18]. TRAMINER showed an improved scar-to-blood signal intensity ratio and a maintained scar-to-myocardium signal intensity ratio, while the myocardium-to-blood contrast was slightly compromised. Although TRAMINER detected two patients with enhanced scar regions that were missed by conventional LGE, no significant difference in the transmural extent of enhanced myocardial segments was found. Additionally, TRAMINER showed non-uniform blood suppression between the RV and LV chambers due to the presence of some T_2_ effects in the used preparation pulses. More recently, the image quality and reliability of TRAMINER was evaluated [19]. In terms of tissue contrast, both subjective and quantitative analysis showed similar results as in their previous 2017 paper. In contrast with previous findings, it was found that scar transmurality was underestimated using TRAMINER. Additionally, it was reported that a possible underestimation of microvascular obstructions may occur when using TRAMINER.

### Preparation with multiple inversion pulses

Instead of using an additional preparatory module before or after the standard inversion pulse, repetitive inversion pulses can be used to simultaneously null multiple tissues after contrast administration. These inversion pulses can be applied either selectively, where only tissues within the imaging slice are affected, or non-selectively, where the entire volume (including the blood pool) is affected.

**Fig. 8:**
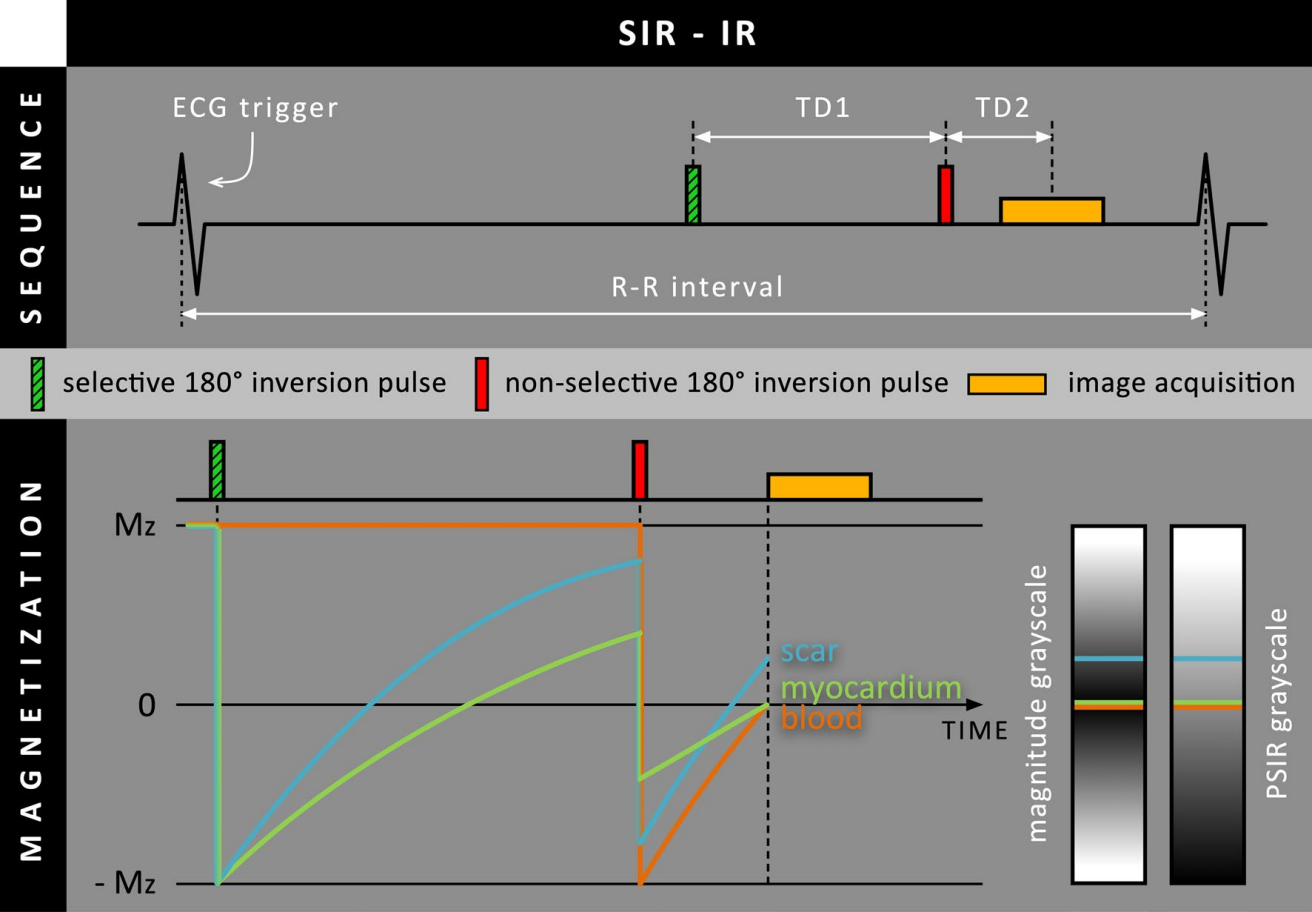
Schematic overview of the SIR-IR method, where a selective 180º inversion RF pulse is performed before the standard (non-selective) 180º inversion RF pulse. Note that the inversion time is split into two delay parameters (TD1 & TD2). For the magnetization diagram, it is assumed that before image acquisition, blood present in the imaging slice at the time of the first (selective) 180º inversion RF pulse has been completely replaced by unaffected blood outside the imaging slice

In 2011, Farrelly et al. proposed an LGE approach that uses two subsequent inversion pulses to simultaneously suppress both normal myocardium and the blood pool [20]. By first applying a selective inversion pulse, both the normal myocardium and area of infarction are inverted while the blood pool remains unaffected due to the inflow of fresh blood (Fig. 8). After a specific delay time (TD1), a non-selective inversion pulse follows, inverting not only the normal myocardium and area of infarction again, but now also the entire blood pool. After another delay (TD1), signal acquisition will take place. Due to the extra selective inversion-recovery (SIR) preparation, we will refer to this approach as SIR-IR.

The SIR-IR approach was first validated in three swine with induced MI, followed by an evaluation in 26 patients with MI [20]. Instead of providing a single TI (as in conventional LGE), the operator had to provide the system with two TIs obtained from the preceding Look-Locker scan: one to null the normal myocardium and one to null the blood pool. The system then automatically calculated the two required delay times. Their results showed that SIR-IR could simultaneously null both the blood pool and normal myocardium, rendering both tissues black with only the areas of MI appearing hyperintense. A 90% increase in scar-to-blood contrast was observed compared to conventional LGE. Furthermore, more foci of grade 1 hyperenhancement (1–25% transmural thickness) were found using SIR-IR compared to standard IR (17 vs 10 foci). Although scar-to-blood contrast improved, a 64% decrease in both scar-to-myocardium contrast and the signal-to-noise (SNR) ratio of the infarcted area was found. A similar trend was observed in the swine model. Furthermore, as this method relies on sufficient blood flow between the selective inversion RF pulse and image acquisition, it was found to work less reliably in patients with an LV ejection fraction < 40% where blood flow is reduced.

**Fig. 9:**
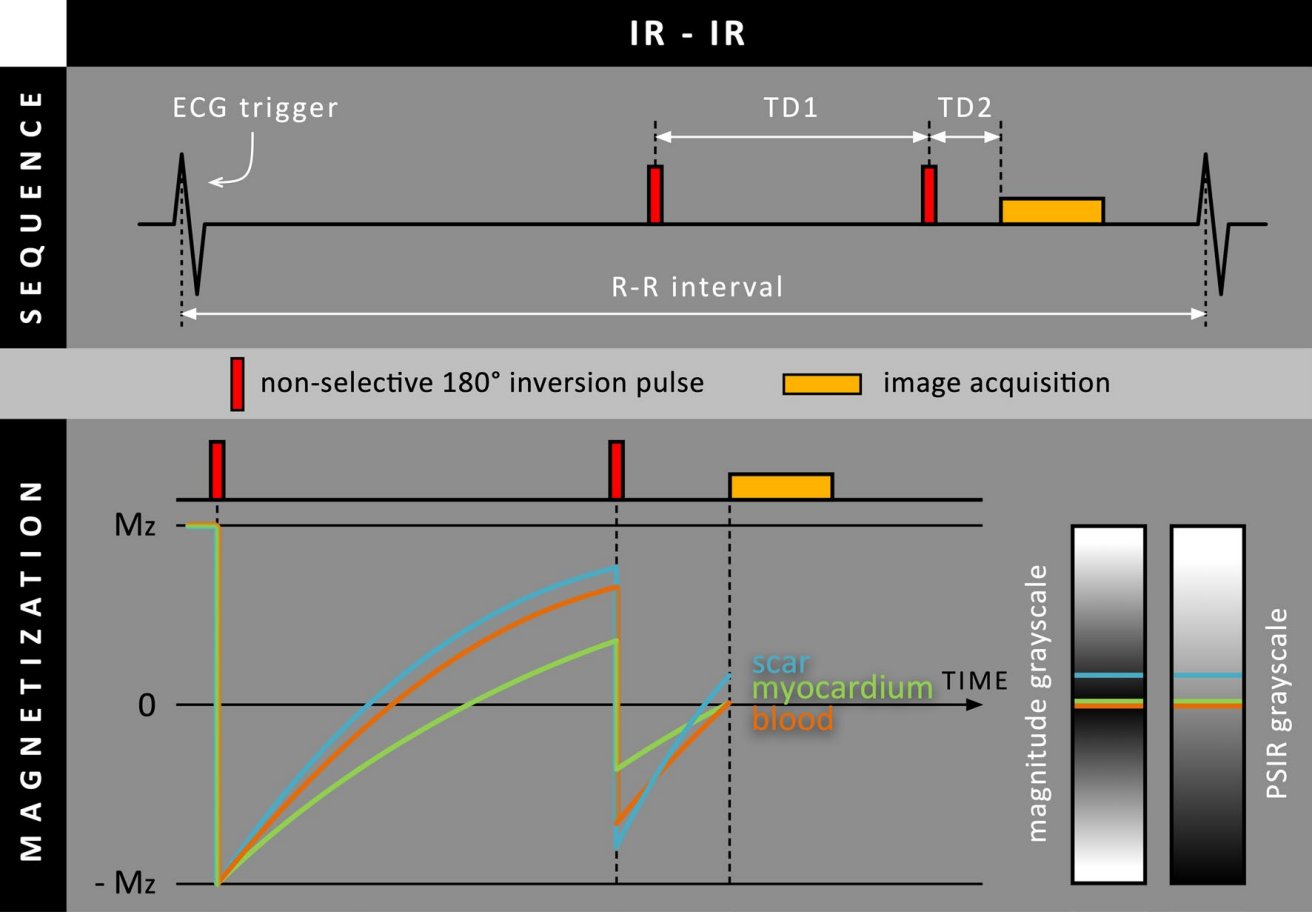
Schematic overview of the IR-IR method, where an additional non-selective 180º inversion RF pulse is performed before the standard non-selective 180º inversion RF pulse. Note that the inversion time is split into two delay parameters (TD1 & TD2)

Later in 2012, Peel et al. proposed an LGE approach that also uses two inversion pulses [21], however, both inversion pulses were applied non-selectively (IR-IR) to render this method insensitive to blood flow (Fig. 9). Using simulations and phantom measurements the TDs were optimized to suppress multiple tissues within four different ranges of T_1_ values (50, 100, 200, 300–1400 ms). Twelve patients with known MI were included and imaged with both conventional LGE and proposed IR-IR LGE. The results showed that IR-IR LGE, compared to conventional LGE, was able to achieve superior scar-to-blood contrast and increased confidence scores for presence of MI. Also, increased consistency between two experts for the assessment of scar transmurality and scar size was demonstrated. However, compared to conventional LGE, both scar-to-myocardium and myocardium-to-blood contrast were significantly reduced. Furthermore, when specific minimal T_1_ values for tissue suppression (50 and 100 ms) were used, scar SNR was reduced compared to conventional LGE.

### No additional magnetization preparation

**Fig. 10:**
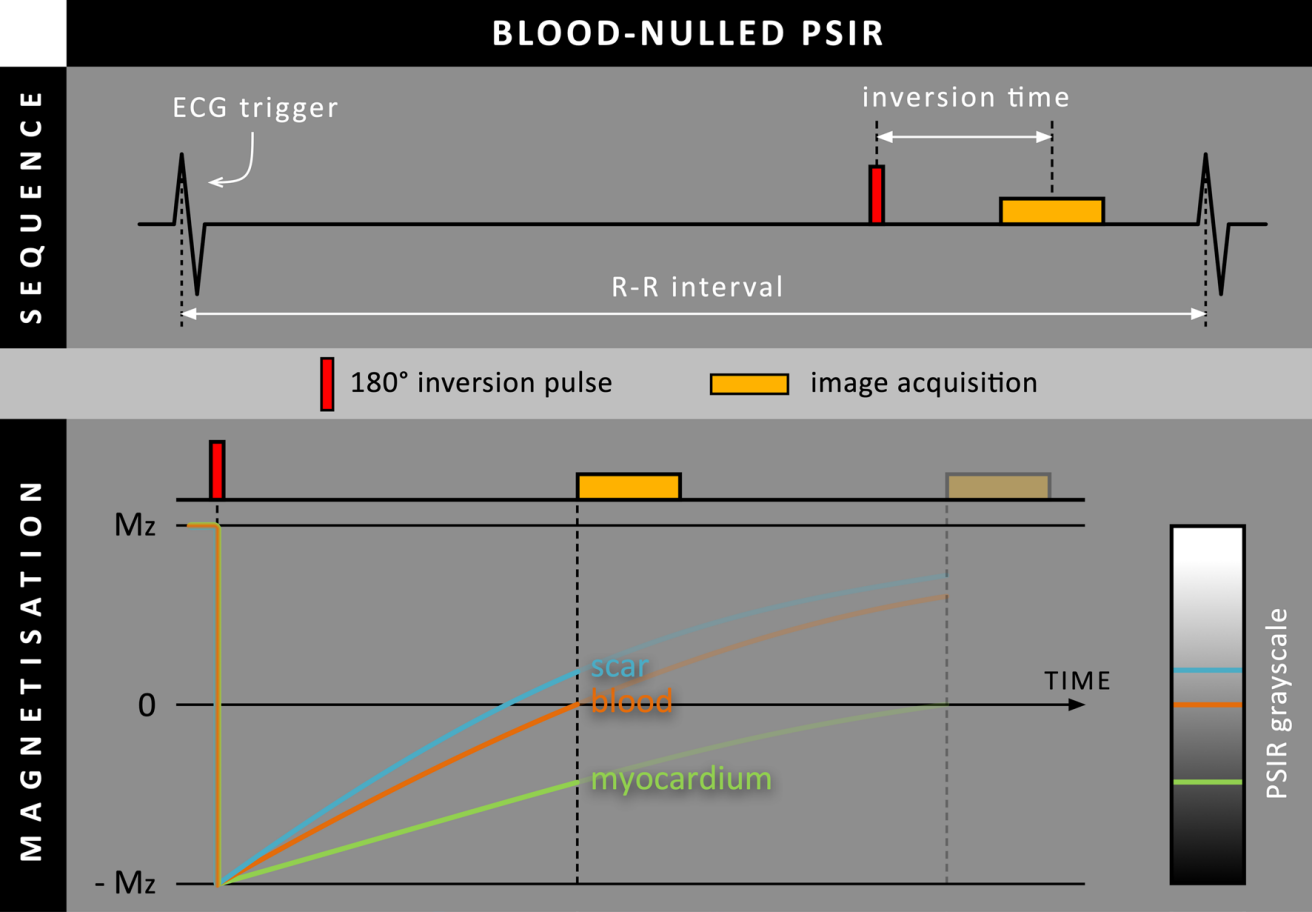
Schematic overview of a standard phase sensitive inversion recovery (PSIR) LGE sequence where the inversion time is set for blood nulling instead of normal myocardium nulling. Note that only the PSIR grayscale is shown for this method, as only the PSIR images are used for clinical decision making. The faded parts of the diagram indicate the situation for conventional bright-blood LGE, where the TI is set for normal myocardium nulling

Although all previous methods used additional magnetization preparation mechanisms to increase scar-to-blood contrast, similar effects can be achieved by shortening the TI to the point of blood pool nulling in a standard PSIR sequence (Fig. 10). Although the blood pool magnetization is nulled and appears black in the magnitude image, it appears dark-gray in the PSIR image as normal myocardium has even lower (negative) magnetization due to its longer T_1_ relaxation time. The PSIR reconstruction mechanism is crucial as it reveals the negative magnetization of normal myocardium and makes it appear black in the resulting PSIR image (instead of bright in the modulus image).

In 2017, Holtackers et al. investigated the feasibility of this blood-nulled PSIR LGE approach without additional magnetization preparation in a small cohort of nine patients with MI and compared it to conventional PSIR LGE [22]. A dedicated noise scan without RF pulses was performed in each patient to enable accurate SNR and CNR measurements, showing a 99% increase in scar-to-blood contrast for blood-nulled LGE compared to conventional myocardium-nulled LGE, regardless of which method was used first. While scar-to-myocardium contrast was maintained, the myocardium-to-blood contrast decreased by 34% compared to conventional LGE. Numerical simulations illustrated the magnetization evolutions towards and during acquisition, which were significantly different for blood nulling than for conventional myocardium nulling, contributing to the observed contrast differences.

Later in 2019, the blood-nulled PSIR LGE approach was validated in an unselected cohort of 300 consecutive patients who were randomly allocated to either a 1.5 T or 3 T scanner of different vendors [23]. Of the 97 patients with ischemic scar tissue on blood-nulled PSIR LGE, eight patients (8.3%) were missed using conventional myocardium-nulled LGE and thus declared free of scar. This effect was observed regardless of which method was acquired first, and regardless of scanner field strength and vendor. Blood-nulled PSIR LGE showed significantly higher observer confidence and intra- and inter-observer agreement, and a significant 10% increase in total scar burden when compared to conventional LGE. As the blood pool appears dark-gray instead of black, blood-nulled PSIR LGE was able to detect all cases of intra-cardiac thrombus. It should be noted that no distinction can be made between the blood pool and scar tissue, in case both have an identical relaxation time.

Also in 2019, Foley et al. performed a study in which this blood-nulled PSIR LGE was compared against T_1_-rho FIDDLE and conventional LGE in a cohort of thirty patients with confirmed prior MI [24]. While both blood-nulled LGE and T_1_-rho FIDDLE showed increased scar-to-blood contrast, only blood-nulled LGE was able to simultaneously maintain, and even exceed, scar-to-myocardium contrast compared to conventional LGE. Additionally, blood-nulled LGE demonstrated significantly higher reader confidence scores compared to both conventional bright-blood LGE and T_1_-rho FIDDLE.

Later, in 2020, the application of blood-nulled PSIR LGE was evaluated in a free-breathing 3D approach with high isotropic resolution (1.6 × 1.6 × 1.6 mm^3^) [25]. As such acquisitions come with longer scan duration, the ideal TI to null the blood pool and obtain dark-blood contrast gradually increases due to continuous contrast washout. Therefore, a steadily increasing dynamic TI mechanism was developed to compensate for contrast washout and optimize contrast. This novel TI mechanism was evaluated in 50 patients and showed significantly better blood pool suppression compared to a conventional fixed TI. As a result, scar demarcation, observer confidence, and overall image quality significantly increased.

More recently in 2021, blood-nulled PSIR LGE was validated against histology in a porcine animal model with experimentally induced MI [26]. Although Bland-Altman analyses demonstrated high levels of agreement with histology for both LGE methods, conventional LGE showed a small but significant bias of -1.57%. In contrast, dark-blood LGE showed no significant bias when compared against histology (-0.03%). CNR analysis demonstrated a significant increase in scar-to-blood contrast for dark-blood LGE compared to conventional LGE, both at 1-week (167%) and 7-weeks (106%) post-MI.

## Discussion and future outlook

Multiple dark-blood LGE methods have been described that suppress or null the blood pool signal while maintaining the bright signal from scar tissue (Table 1). Although mostly desired for improved detection of subendocardial scar areas, dark-blood LGE methods may also be beneficial for visualizing scar patterns in papillary muscles and thin-walled structures, such as the atria and RV.

**Table 1:** Overview of proposed dark-blood LGE methods

Magnetization preparation	Method name *(acronym)*	Year	Number of subjects	Field strength	Blood M_z_ < Myo M_z_	Blood appearance	Myocardium appearance	S-B CNR^a^	S-M CNR^a^	M-B CNR^a^	Reference standard
T_2_ preparation	T_2_ prep-IR *(no PSIR)*	2008 [7]	9 patients 5 controls	1.5 T	No	Dark-gray	Black	↑	○	↓	Standard LGE^b^
	T_2_ prep-IR *(FIDDLE)*	2020 [15]	35 patients	1.5 T + 3 T	Yes	Black	Gray	↑↑	↓	↓	None
	IR-T_2_ prep	2016 [8]	61 patients no controls	1.5 T	Yes	Black	Dark-gray	↑↑	↓	↓	Standard LGE^b^
		2017 [9]	172 patients no controls	1.5 T	Yes	Black	Dark-gray	↑↑	↓	↓	Standard LGE^b^
		2018 [10]	9 pigs 42 patients no controls	1.5 T	Equal	Black	Black	↑↑	↓	Absent	Standard LGE^b^
		2018 [11]	5 swine 45 patients no controls	1.5 T	Equal	Black	Black	↑↑	↓	Absent	Standard LGE^b^
					Yes	Dark-gray	Black	↑	○	↓	
Magnetization transfer	MT-IR *(FIDDLE)*	2018 [14]	22 dogs 20 patients 11 controls	1.5 T + 3 T	Yes	Black	Gray	↑↑	↓	↓	Histology + Standard LGE^b^
Spin-locking (+ MT)	SL/MT-IR *(TRAMINER)*	2017 [18]	40 patients no controls	1.5 T	Yes	Black	Dark-gray	↑↑	○	↓	Standard LGE^b^
		2019 [19]	73 patients no controls	1.5 T	Yes	Black	Dark-gray	↑↑	↓	↓	Standard LGE^b^
Multiple inversion pulses	SIR-IR	2011 [20]	3 swine 26 patients no controls	1.5 T	Equal	Black	Black	↑↑	↓	Absent	Standard LGE^b^
	IR-IR	2012 [21]	15 patients no controls	3 T	Yes	Dark-gray	Black	↑	↓	↓	Standard LGE^b^
No additional magnetization preparation	Blood-nulled PSIR	2017 [22]	9 patients no controls	1.5 T	No	Dark-gray	Black	↑	○	↓	Standard LGE^b^
		2019 [23]	300 patients no controls	1.5 T + 3 T	No	Dark-gray	Black	↑	○	↓	Standard LGE^b^
		2021 [25]	50 patients no controls	1.5 T	No	Dark-gray	Black	↑	○	↓	Standard LGE^b^
		2021 [26]	13 pigs	1.5 T	No	Dark-gray	Black	↑	○	↓	Histology + Standard LGE^b^

^a^Indicated values are relative to conventional (bright-blood) LGE with normal myocardium nulling: ↑ = increased, ↓ = decreased, ○ = maintained

^b^Conventional (bright-blood) LGE with normal myocardium nulling

CNR = contrast-to-noise ratio, IR = inversion-recovery, M-B = myocardium-to-blood, MT = magnetization transfer, M_z_ = longitudinal magnetization, PSIR = phase-sensitive inversion-recovery, S-B = scar-to-blood, S-M = scar-to-myocardium, SIR = selective inversion-recovery, SL = spin-locking

### Blood pool appearance

**Fig. 11:**
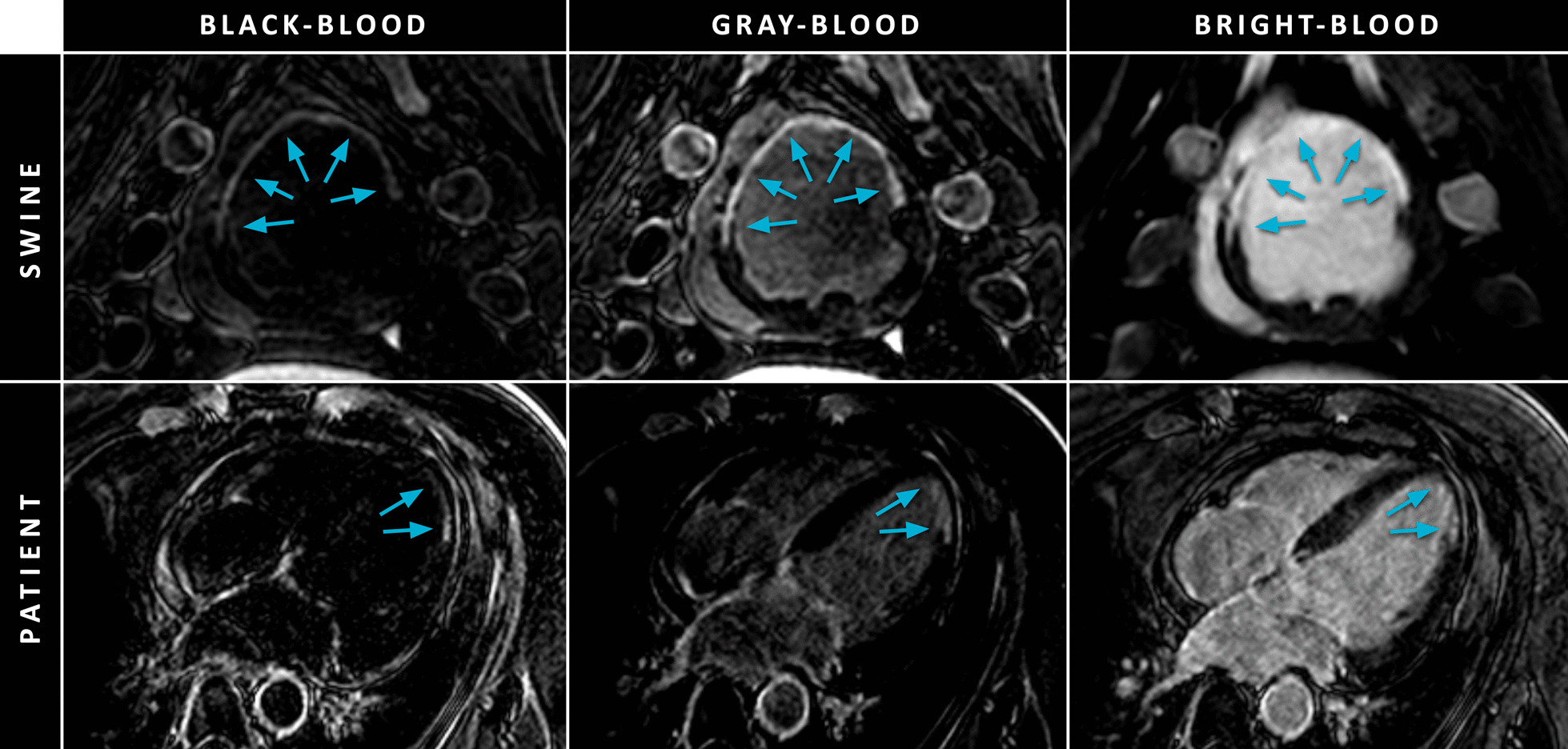
Black-blood (left column), gray-blood (middle column), and conventional bright-blood PSIR (right column) LGE images of a swine (top row, short-axis view) and patient (bottom row, four-chamber view) with MI, indicated by the cyan arrows. Both black-blood and gray-blood images were acquired using the IR-T_2_ prep method, although with different parameter settings, and were adapted from Fahmy et al. [11] with author permission

While these methods share a common goal, the methodologies to do so vary greatly. The appearance of the blood pool in the final image determines whether it is a black-blood or gray-blood method, in contrast to the conventional bright-blood LGE method (Fig. 11).

Black-blood methods are able to achieve excellent contrast between the black blood pool and bright scar tissue. The normal myocardium, however, often appears (dark-)gray instead of black (in conventional LGE). As a result, the scar-to-myocardium contrast is regularly compromised compared to conventional bright-blood LGE, potentially lowering the sensitivity for non-ischemic scar tissue. Other black-blood methods aim to simultaneously null both the blood pool and normal myocardium, thereby maintaining scar-to-myocardium contrast required for non-ischemic scar detection. With these particular methods, however, the myocardium-to-blood contrast required for anatomical reference is significantly reduced, potentially preventing one from assessing the exact location and transmurality of any bright area of scarring. Regardless of the appearance of the normal myocardium in black-blood LGE methods, the detection of intracardiac thrombi is compromised compared to conventional LGE as they appear equally black as the blood pool. However, by adjustment of specific sequence parameters, most black-blood methods can be adapted to gray-blood techniques [11].

**Fig. 12:**
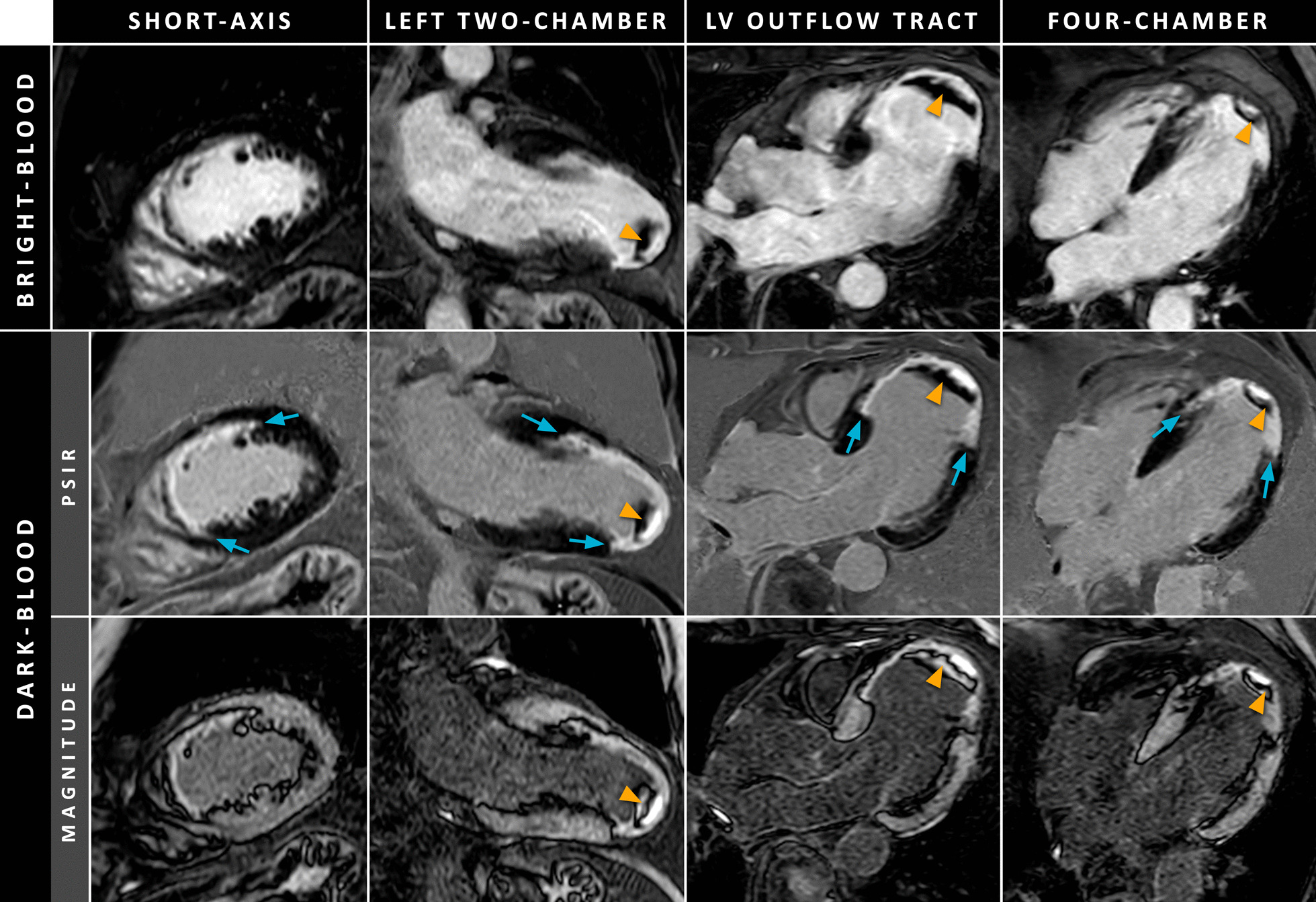
Imaging example of thrombus appearance using conventional bright-blood (upper row) and dark-blood (middle row) PSIR LGE in multiple cardiac views in a patient with prior MI. The lower row illustrates the corresponding magnitude images of the middle row (where the inversion time was set for blood pool nulling). Note that for this dark-blood LGE method, only the PSIR dark-blood images are used for clinical decision making. The cyan arrows indicate the areas of MI, while the orange arrowheads indicate the intracardiac thrombus. The dark-blood images were acquired using the blood-nulled PSIR LGE method and were adapted from Holtackers et al. [23] with author permission

On the other hand, gray-blood LGE methods are also able to achieve improved scar-to-blood contrast. As the blood pool is not completely black, the scar-to-blood contrast is usually not as high as in black-blood methods, however, still significantly increased compared to conventional bright-blood LGE. For most gray-blood methods, the normal myocardium appears equally black as in conventional LGE, thereby resulting in maintained or only slightly decreased scar-to-myocardium contrast. Since the blood pool appears darker than in conventional LGE, the myocardium-to-blood contrast is decreased, however, still adequate for anatomical reference. Additionally, as the blood pool is appearing gray instead of black, the detection of intracardiac thrombi is maintained using gray-blood LGE (Fig. 12) [23].

Besides the blood pool appearance, there is a fundamental difference between techniques that reduce the blood pool signal below that of normal myocardium versus those that result in blood pool signal that is still above normal myocardium. This distinction is important, for example in cases of patchy subendocardial scarring where the post-contrast T_1_ relaxation time is longer than that of blood. For methods where the blood pool signal is still above normal myocardium, the signal of this patchy subendocardial scarring will be in between that of normal myocardium and blood, and may resemble that of the blood-myocardium interface, which might render it invisible. For methods where the blood pool signal is below that of normal myocardium, the signal of patchy scarring will be in between that of normal myocardium and (dense) scarring. Although less clearly visible, patchy scarring will still be detectable. Table 2 provides an overview of the key differences between black-blood and gray-blood LGE, with conventional bright-blood LGE methods as a reference.

**Table 2:** Black-blood versus gray-blood LGE with conventional bright-blood LGE as a reference

	Black-blood LGE	Gray-blood LGE
Scar-to-blood contrast	↑ ↑	↑
Scar-to-myocardium contrast	○ / ↓^a^	○
Myocardium-to-blood contrast	↓ ↓ / ↓^a^	↓
Assessment of scar transmurality	○ / ↑^a^	↑
MI size quantification	↑ ↑ / ↑^a^	↑
MVO detection	○^b^	○^b^
LV thrombus detection	↓ ↓	○

Indicated values are relative to conventional (bright-blood) LGE with normal myocardium nulling: ↑ = increased, ↓ = decreased, ○ = maintained

^a^First value indicative for black-blood methods that simultaneously null both blood pool and normal myocardium (both appear black), second value indicative for black-blood methods with a dark-gray appearance of the normal myocardium

^b^Equal detection but might improve differentiation with thrombus

LGE = late gadolinium enhancement, LV = left-ventricular, MI = myocardial infarction, MVO = microvascular obstruction

### Practical clinical utility

The improved detection and visibility of subendocardial scar patterns make dark-blood LGE methods a useful tool in clinical routine settings. A 2021 study by Franks et al. showed that dark-blood LGE detects a higher ischemic scar burden than conventional bright-blood LGE and leads to a lower estimation of the myocardial ischemic burden when used in conjunction with perfusion imaging [27]. This can lead to disagreement around established thresholds of clinically significant ischemia used for revascularization decision making. However, since most studies focused on the detection of ischemic scar tissue, the diagnostic performance of these methods for detecting non-ischemic scar patterns remains largely unknown. Although multiple studies showed a decrease in scar-to-myocardium contrast, which in theory may hamper the detection of non-ischemic scar patterns compared to conventional bright-blood LGE, no general conclusions can be drawn yet. Even though dark-blood LGE methods may already replace conventional LGE in specific settings (e.g. ischemic heart disease and myocardial infarction with non-obstructive coronary arteries (MINOCA)), they remain as addition to conventional LGE in generalized cardiomyopathy scan protocols.

### Ease of use

Compared to the single TI used for conventional LGE, most magnetization preparation schemes come with additional imaging parameters. These parameters include additional delays, durations, and specific RF pulse settings, such as the flip-angle, B_1_ strength, off-resonance frequency, and pulse train length and phase. Some have to be set only once when implementing and setting up the protocol, while others need to be adjusted individually for each patient. For some methods, this may require an additional T_1_ map [8, 9, 11] or contrast-scout scan [10] prior LGE imaging to calculate the optimal delay parameters. Other methods require repetitive pre-scans to empirically derive the optimal TI [18]. These requisites may require additional training and can prolong scan duration.

Apart from acquisition, the new types of image contrast may also introduce additional training for readers assessing these images. Techniques where both the blood pool and normal myocardium are nulled may require co-registration with cine images to determine and assess the myocardial borders. For methods that use additional magnetization preparation modules, readers need to be familiar with the T_2_, MT, and T_1_-rho effects on the cardiac structures and their appearance in various cardiomyopathies to assure accurate analysis of the underlying pathologies.

### Availability

Although the superiority of dark-blood LGE methods in detecting (sub)endocardial scar tissue compared to conventional LGE is already proven, clinical translation of dark-blood LGE is not straightforward. Most methods use additional magnetization preparation schemes that are not available in a commercial CMR system configuration. Software patches or work in progress (WIP) packages are required to perform those new preparation schemes on clinical CMR systems. Even though these may be made available by the vendors or have been implemented in individual centers for research purposes, legal regulations may prevent their use in routine patient care, hampering widespread clinical implementation. Methods without additional magnetization preparation mechanisms, however, are already readily and widely available on clinical CMR systems.

### Scar quantification methods

The improved scar-to-blood contrast achieved by dark-blood LGE methods may benefit the delineation and quantification of ischemic scar patterns. Kim et al. already showed that manual delineation of ischemic scar using dark-blood LGE led to significantly better sensitivity and accuracy (96 and 95%, respectively) than using conventional bright-blood LGE (85 and 87%, respectively) [14]. Differences in sensitivity and accuracy further increased when only slices with < 25% transmural infarction were considered (98 and 95% vs 80 and 85%, respectively). Although Foley et al. found a 37.5% larger transmural extent of scar using dark-blood LGE compared to conventional LGE, no significant difference between both techniques was reported when using the full-width half-maximum (FWHM) quantification method [24]. This method, however, was found to under-estimate dark-blood LGE scar size by over 25% [28]. Instead, using manual contouring as reference, the signal threshold versus reference mean method with a 5-standard deviation (SD) threshold most accurately quantified infarct size on dark-blood LGE images, thereby outperforming the 6-SD, FWHM, and the Otsu auto-threshold methods. To the best of our knowledge, no semi-automatic quantification methods have been validated against histopathology for dark-blood LGE. As scar quantification is becoming increasingly important in recent years, future research should focus on validating quantification methods for dark-blood LGE to evaluate clinical benefit.

### Phase-sensitive inversion-recovery

The majority of dark-blood LGE methods use a PSIR sequence to reveal negative magnetization levels during signal readout. Without PSIR, these magnetization levels would be visualized similarly to positive magnetization levels of the same magnitude, potentially decreasing tissue contrast. PSIR therefore makes LGE image quality less sensitive to the chosen TI, leading to a reduction in image artefacts and potential misinterpretations. Additionally, in contrast to standard IR, PSIR is a two-beat sequence making it more robust to heart rate variations and cardiac arrhythmias as it relies to a lesser extent on a constant time delay between successive inversion RF pulses, averaging irregular heartbeats over two heart beats. On the other hand, however, mismatches between the image (first heartbeat) and reference (second heartbeat) readout may lead to suboptimal image quality and scan duration inherently doubles when using PSIR instead of standard IR.

### Field strength dependency

Although LGE CMR can be performed on both 1.5 and 3 T scanners, there are limited data for many of the dark-blood methods at 3 T  (Table 1) as most were proposed and validated on 1.5 T. The increased field strength of 3 T may influence the performance of the additional preparation modules required for most dark-blood methods. As demonstrated in a recent study by Jenista et al., T_2_ preparation at 3 T was prone to more in-flow artifacts in the left atrium and increased differences in RV-to-LV blood-pool suppression compared to 1.5 T [15]. Also, when using MT preparation, more in-flow artefacts were observed at 3 T compared to 1.5 T. However, no visible differences in blood suppression between the LV and RV were observed for MT preparation at both 1.5 T and 3 T. With only a few dark-blood methods (also) evaluated at 3 T [14, 15, 21, 23], other methods, in particular those using field strength dependent magnetization preparation modules, should also be evaluated at 3 T to assess their clinical utility.

### Future research

While most novel dark-blood methods are compared to conventional LGE, only FIDDLE and blood-nulled PSIR LGE have been validated against histology [14, 26], and direct comparison studies evaluating different dark-blood LGE methods are limited [24]. Such comparison studies are mostly hindered by the limited availability of most techniques. Ideally, more direct comparison studies should be conducted to evaluate the individual performance of the various dark-blood LGE methods compared to conventional LGE with appropriate dose and timing [29]. Additionally, their effect on (semi-)automatic scar quantification methods should be investigated.

Exciting new avenues that hold promise for dark-blood LGE include the combination with image-navigated free-breathing 3D acquisitions with high isotropic resolution. 2D image-based navigators directly track the position of the heart itself and can correct for translational motion to enable 100% efficiency and thus more predictable scan durations [30]. Although the feasibility of 2D image-based navigators for conventional free-breathing 3D LGE has been investigated [31, 32], future work should focus on the implementation in dark-blood LGE approaches. The recent introduction of compressed sensing by the major vendors enabled widespread use of sparse imaging techniques, achieving acceleration factors that have not previously been possible to attain with parallel imaging alone. Additionally, artificial intelligence-based CMR reconstruction techniques may be used to further enhance the use of compressed sensing methods [33].

## Conclusions

Dark-blood LGE methods are a promising new tool for non-invasive assessment of myocardial infarction. Although the discussed mechanisms improve detection of subendocardial scar, their weaknesses in terms of scar-to-myocardium contrast, blood appearance, availability, and ease of use, vary significantly. For a mainstream adoption of dark-blood LGE methods, however, clinical availability and ease of use are crucial.

## Data Availability

Not applicable.

## Abbreviations

BIR-4: B_1_-insensitive rotation-4
CMR: Cardiovascular magnetic resonance
CNR: Contrast-to-noise ratio
DE: Delayed enhancement
FIDDLE: Flow independent dark-blood delayed enhancement
FWHM: Full-width half-maximum
IR: (Non-selective) inversion-recovery
LE: Late enhancement
LGE: Late gadolinium enhancement
LV: Left ventricle/left ventricular
MCODE: Multi contrast delayed enhancement
MI: Myocardial infarction
MT: Magnetization transfer
M_xy_: Transversal magnetization
M_z_: Longitudinal magnetization
MVO: Microvascular obstruction
PSIR: Phase-sensitive inversion-recovery
RF: Radiofrequency
RV: Right ventricle/right ventricular
SIR: Selective inversion-recovery
SD: Standard deviation
SL: Spin-locking
SNR: Signal-to-noise ratio
TE_eff_: Effective echo time
TI: Inversion time
TRAMINER: Transfer and inversion recovery prepared imaging
WIP: Work in progress
